# Determining the structure of a benzene7.2-silicalite-1 zeolite using a single-crystal X-ray method

**DOI:** 10.1107/S0108768111038560

**Published:** 2011-10-13

**Authors:** Natsumi Kamiya, Wataru Iwama, Tomokazu Kudo, Tomomi Nasuno, Shinjiro Fujiyama, Koji Nishi, Yoshinobu Yokomori

**Affiliations:** a Department of Applied Chemistry, National Defense Academy, Hashirimizu, Yokosuka 239-8686, Japan

**Keywords:** ZSM-5, MFI, silicalite-1, benzene-silicalite-1

## Abstract

A simple method for preparing orthorhombic single crystals of benzene-silicalite-1 was developed. A silicalite-1 crystal was pressed with a weight of 2 g along the +*c* and −*c* crystallographic axes while the temperature was increased to 473 K. The temperature was then slowly reduced to 313 K, and these heating and cooling steps were repeated three times. After the orthorhombic single crystals adsorbed benzene, the crystal structure of the resulting benzene-silicalite-1 was determined. There were two kinds of benzene molecules in the asymmetric unit. One was located at the intersection of the straight channels and the sinusoidal channels with the benzene ring parallel to the *ac* plane. The other benzene was located in the middle of the straight channel.

## Abstract 2

An orthorhombic benzene-silicalite-1 single crystal was obtained from a monoclinic twin crystal, and the structure was determined by a single-crystal method for the first time.

## 1. Introduction

The aluminosilicate ZSM-5 and silicalite-1, a high silicate zeolite, have attracted considerable recent interest due to their wide applicability as shape-selective catalysts and adsorbents. Many aromatic sorbate-ZSM-5 and sorbate-silicalite-1 structures have been investigated by single-crystal X-ray diffraction (van Koningsveld, Tuinstra, van Bekkum & Jansen, 1989 ▶; Reck *et al.*, 1996 ▶; van Koningsveld, Jansen & de Man, 1996 ▶; van Koningsveld, Jansen & van Bekkum, 1996 ▶; Nishi *et al.*, 2005 ▶, 2007 ▶). However, the benzene-ZSM-5 and benzene-silicalite-1 structures have not yet been determined by single-crystal X-ray diffraction.

**Figure 1:**
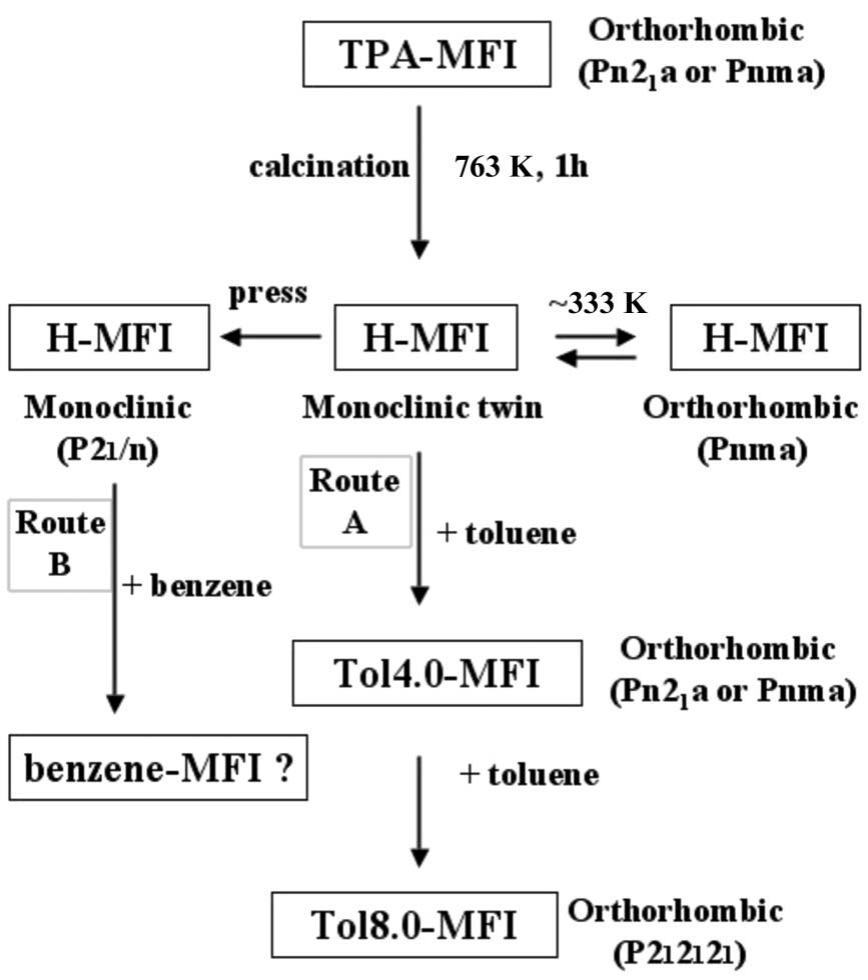
Phase transitions of MFI zeolite.

**Figure 2:**
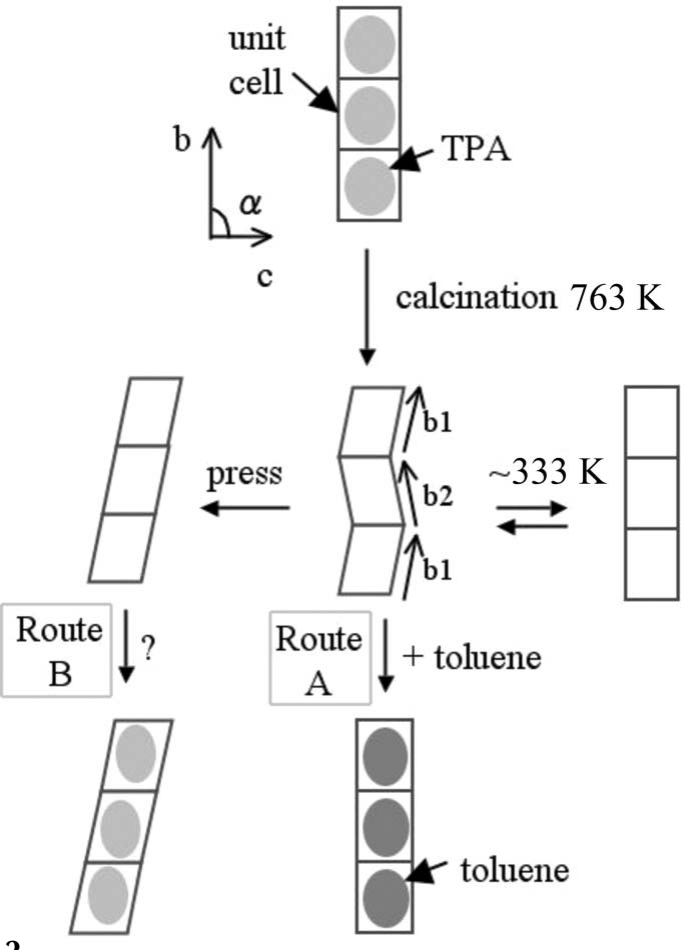
Model of the phase transitions of MFI zeolite.

ZSM-5 and silicalite-1, both MFI (IUPAC code of this family) zeolites, undergo many phase transitions with calcinations or adsorption, as summarized in Fig. 1 ▶. A model of these phase transitions is shown in Fig. 2 ▶. Initially, the orthorhombic crystal phase of as-synthesized tetrapropylammonium (TPA)-MFI zeolite transforms into monoclinic twin phases after calcination. The monoclinic twin crystal, H-ZSM-5, exhibits a reversible phase transition to a single-crystal orthorhombic phase at ∼ 340 K (van Koningsveld, Jansen & van Bekkum, 1987 ▶). On the other hand, van Konigsveld *et al.* obtained a single crystal of monoclinic ZSM-5 after applying uniaxial mechanical stress that altered the populations of the monoclinic twin domains (van Koningsveld, Tuinstra, Jansen & van Bekkum, 1989 ▶). They also analyzed the single-crystal structure of monoclinic ZSM-5 (van Koningsveld, Jansen & van Bekkum, 1990 ▶). The authors recently developed a simple method for preparing monoclinic single crystals of ZSM-5 and determining the monoclinic structure of ZSM-5 (Kamiya *et al.*, 2010 ▶). Generally, monoclinic twin MFI crystals transform into orthorhombic sorbate-MFI single crystals after adsorbing aromatic compounds other than benzene. However, after adsorbing benzene or chain compounds, the crystals remain in the monoclinic twin phase, so the structures of benzene-ZSM-5 and benzene-silicalite-1 remain unclear.

In this report the authors present a new method of obtaining single crystals of benzene-silicalite-1, and describe its structure, which was determined for the first time by a single-crystal method.

## 2. Experimental

### 2.1. Preparation of tetrapropylammonium-silicalite-1

Crystals of TPA-silicalite-1 were synthesized using the method described by Kamiya *et al.* (2007 ▶). The mixture had the following molar composition: 12SiO_2_:34KOH:40TPABr:2000H_2_O. The quantity of KOH was reduced to obtain better crystals, as described in Kamiya *et al.* (2007 ▶). The crystals were synthesized using silicalite-1 (0.7 wt% of SiO_2_) seeds for 7 d at 453 K. Approximately 10 d were required to obtain good crystals without a seed. The obtained samples were washed with distilled water and dried at 388 K for 24 h.

### 2.2. Sodium perchlorate treatment and calcination

Normally calcination of the crystals to remove TPA ions results in cracking over 80% of the crystals (Geus & van Bekkum, 1995 ▶). A sodium perchlorate treatment was developed by the authors to avoid crystal cracking (Kamiya *et al.*, 2010 ▶). After this treatment, the crystals were calcined at 763 K in flowing air for 1 h to obtain monoclinic twin silicalite-1 crystals.

### 2.3. Preparation of monoclinic single crystals of silicalite-1

The preparation of monoclinic single crystals of silicalite-1 was described in detail in Kamiya *et al.* (2010 ▶).

### 2.4. Preparation of orthorhombic single crystals of silicalite-1

**Figure 3:**
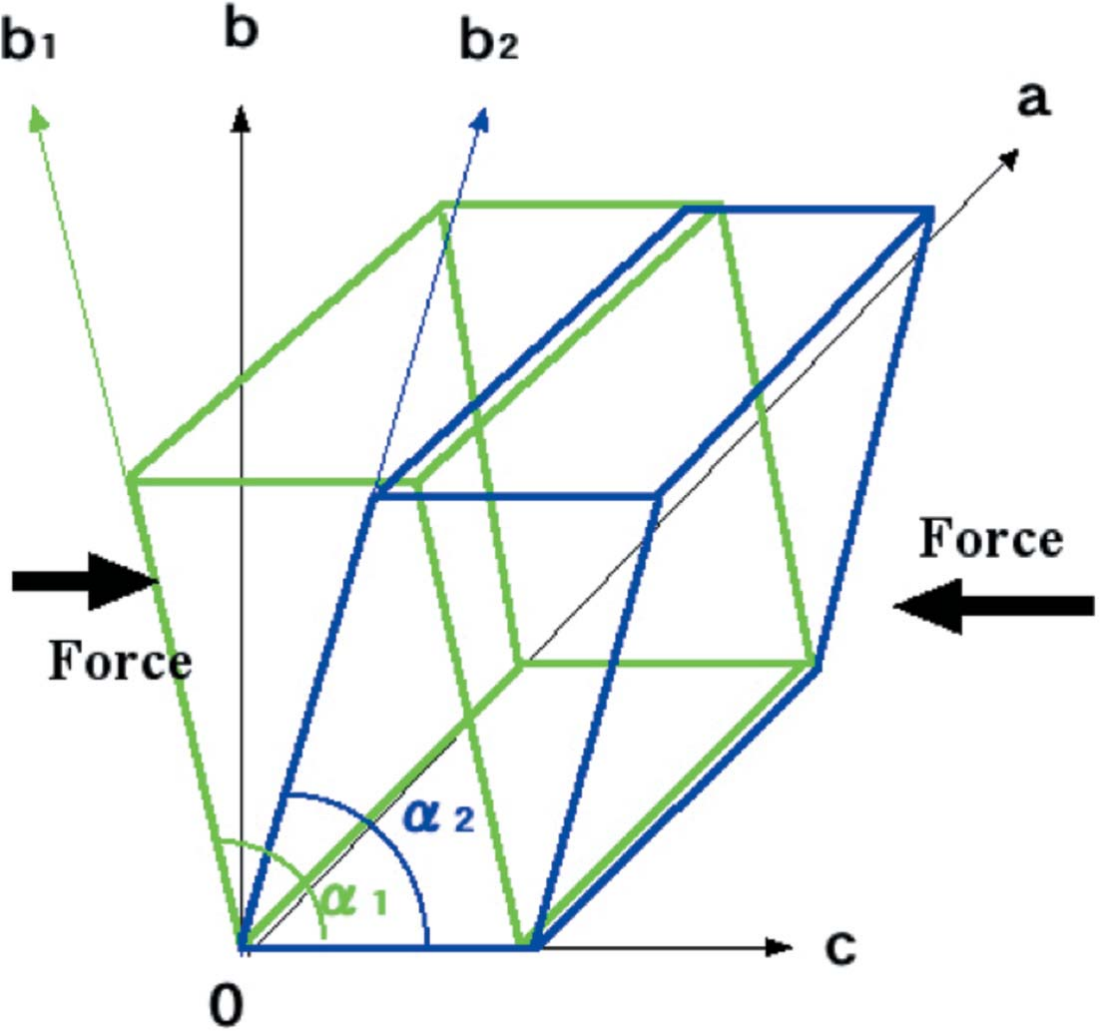
Model of a monoclinic twin silicalite-1 crystal and definitions of α_1_ and α_2_.

A model of a monoclinic twin silicalite-1 crystal is shown in Fig. 3 ▶, along with the crystal parameters (*a*, *b*, *c*, α) and two kinds of α angles (α_1_ + α_2_ = 180°). When the crystal parameters are (*a*_1_, *b*_1_, *c*_1_, α_1_) and (*a*_2_, *b*_2_, *c*_2_, α_2_) in Fig. 3 ▶, their relationships are *a*_2_ = *a*_1_, *b*_2_ = −*b*_1_, *c*_2_ = −*c*_1_, α_2_ = 180 − α_1_. In the case of silcalite-1 and ZSM-5, as the angles of 90 − α_2_ are less than 0.6°, most of the reflections overlap (van Koningsveld, Jansen & van Bekkum, 1987 ▶; van Koningsveld, Tuinstra, Jansen & van Bekkum, 1989 ▶ ▶). The monoclinic twin crystal was pressed along the +*c* and −*c* crystallographic axes (Fig. 3 ▶), while the temperature was increased from 313 to 473 K over 30 min and then cooled to room temperature over ∼ 6 h in the furnace. These heating and cooling steps were repeated three times.

**Figure 4:**
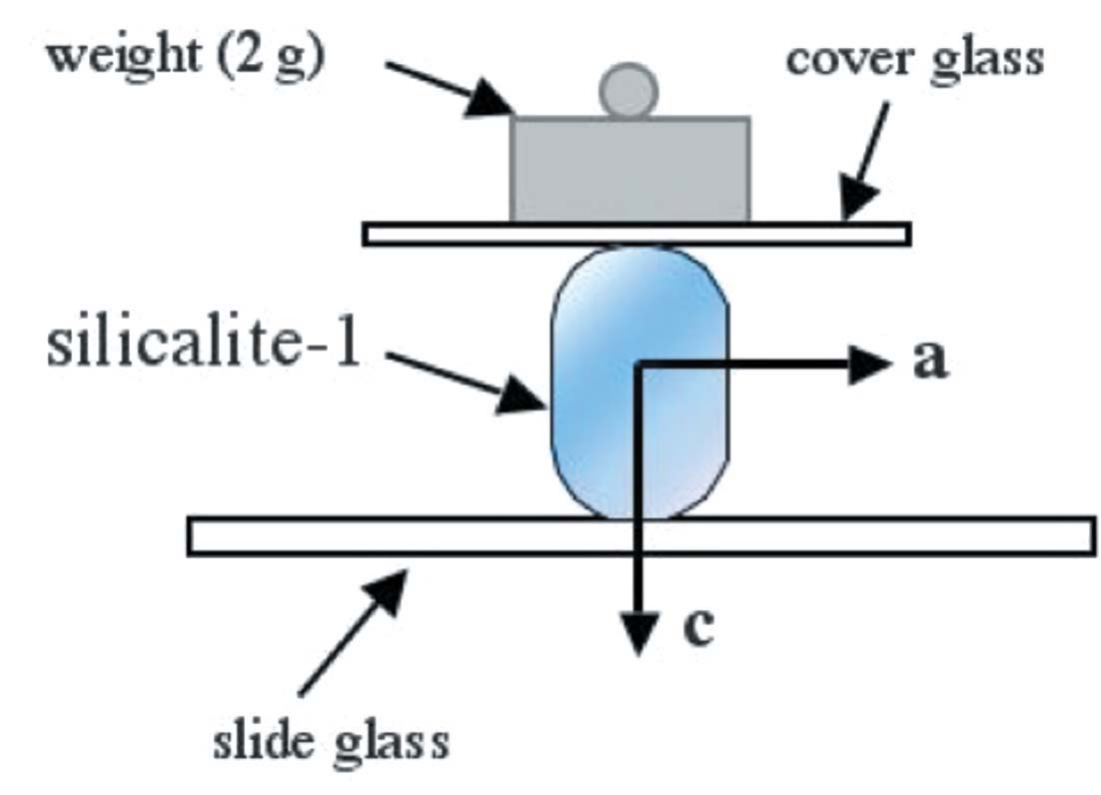
Pressing treatment for the phase transition from the monoclinic twin to the orthorhombic single silicalite-1 crystal.

The crystal geometry of the silicalite-1 and ZSM-5 can be easily understood because the widest crystal face is always the (010) face and the longest straight sides are always parallel to the *c* axis. The crystal was pressed with a weight of 2 g and held between a microscope cover glass and a glass microscope slide without any glue during this process (Fig. 4 ▶). The cover glass size was ∼ 10 mm and the crystal size was less than 0.3 mm, so it was not difficult to position the crystal under the microscope if the crystal position was marked on the slide glass.

The authors assumed that these single crystals were orthorhombic by analogy with the preparation of simple monoclinic silicalite-1 (Kamiya *et al.*, 2010 ▶). The authors confirmed that they were orthorhombic according to the results of the structure analysis of orthorhombic benzene-silicalite-1. This way of preparation is very important because it would be very difficult to obtain any information regarding orthorhombic benzene-silicalite-1 structure without it.

### 2.5. Adsorption of benzene in silicalite-1

A prepared silicalite-1 crystal was exposed in a closed vacuum oven (Bell jar-type vacuum oven BV-001, Shibata Science Co.) to saturated benzene (∼ 13 kPa) at room temperature for 120 h. Thermal gravimetric analysis (TG-DTA2000SA, Bruker AXS) indicated that the crystal consisted of 7.2 benzene molecules per unit cell. The chemical composition related to the unit cell is Si_96_O_192_·7.2C_6_H_6_ by TG-DTA (differential thermal analysis).

### 2.6. X-ray analysis of monoclinic benzene-silicalite-1 structure

Generally, monoclinic twin MFI crystals transform into orthorhombic sorbate-MFI single crystals after adsorbing toluene, *p*-xylene or *p*-dichlorobenzene (Route A in Figs. 1 ▶ and 2 ▶). In the case of benzene, however, no work using single crystals had yet been reported. Recently, a simple monoclinic single-crystal preparation of silicalite-1 was developed by the authors (Kamiya *et al.*, 2010 ▶). After this preparation, these monoclinic silicalite-1 crystals adsorbed benzene, but were twinned. It was difficult to separate the overlapping twin crystals because the angle 90 − α_2_ was less than 0.6° (Fig. 3 ▶). Over 20 crystals were analyzed using X-ray reflections that neglected one twin domain, but the results were unsatisfactory; that is, the direct method did not always work and could not determine even the framework structure. Even when the direct method did work, the best *R* values were larger than 0.12. According to X-ray analysis (van Koningsveld, Jansen & van Bekkum, 1990 ▶), the monoclinic framework is less strained than the orthorhombic framework. After the monoclinic silicalite-1 adsorbs aromatic sorbate, the monoclinic framework becomes less stable than the orthorhombic framework. The larger the size of the aromatic sorbate, the more stable the orthorhombic framework. Since benzene is too small, the benzene-silicalite-1 monoclinic framework cannot completely transform into the orthorhombic framework.

### 2.7. Orthorhombic benzene7.2-silicalite-1 structure

Orthorhombic silicalite-1 crystals were prepared by the method described in §2.4, and benzene was adsorbed onto these crystals for 120 h. Over 20 single crystals of ortho­rhombic benzene-silicalite-1 were analyzed by X-ray diffraction. In many cases the first as-synthesized TPA-silicalite-1 crystals were always of very high quality, but after treatment with sodium perchlorate and calcination (763 K, 1 h), and the preparation of monoclinic and orthorhombic single crystals [(473 K, 30 min) × 3] the crystal quality became very low. X-ray analysis was attempted until crystals of sufficient quality were obtained. The authors did not search for the origin of the low crystal quality, but cracking of the silicalite-1 crystals was observed during calcination (Geus & van Bekkum, 1995 ▶). Of course, the results of structure analysis were always similar to those of good crystals. The best crystal data and refinement details are shown in Table 1 ▶ 1 and the positional parameters have been deposited.

### 2.8. X-ray analysis of orthorhombic benzene7.2-silicalite-1

Single-crystal X-ray diffraction analysis was carried out at room temperature using an APEX II X-ray diffractometer (Bruker AXS) with a CCD detector, Mo *K*α radiation and a graphite monochromator. The crystal selected for X-ray analysis measured 0.26 × 0.14 × 0.12 mm. There were 50 699 reflections collected from the sphere of reflection (*h* −24 to 24, *k* −23 to 23, *l* −16 to 16), and corrected for Lorentz-polarization and absorption effects. The systematic absences (*hk*0, *h* = 2*n* + 1; 0*kl*, *k* + *l* = 2*n* + 1) indicate a space group of *Pnma* or *Pn*2_1_ *a*.

The structures were solved by direct methods (*SHELX* 97 in APEX II; Sheldrick, 2008 ▶), and the difference-Fourier synthesis was used for the remaining atoms. The structure was initially solved in a non-centrosymmetrical space group *Pn*2_1_ *a* in order to avoid possible disorder. Later on the center of symmetry was added and the structure was successfully refined in the space group *Pnma*. After the initial direct method, the *R* value was 0.103 and the difference-Fourier map indicated a silicalite-1 framework and two C atoms of benzene in the straight channel. Isotropic refinement of the only framework gave *R* = 0.114. After a few least-square cycles, the *R* value including the framework and one independent benzene in the straight channel dropped to 0.081 and the difference-Fourier map clearly showed another independent benzene at the intersection. After a few cycles, isotropic refinement of the framework and two independent benzene molecules gave *R* = 0.063 and the corresponding anisotropic refinement converged at *R* = 0.038. During the last few cycles, two independent benzene molecules were restrained to avoid deformation; that is, all of the C atoms in the benzene were constrained to an ideal benzene ring (the C—C bonds were 1.39 Å, and all of the carbon atoms were coplanar). Only one peak (+0.89 e Å^−3^) from the difference-Fourier synthesis located in the sinusoidal channel could not be understood. Although silicalite-1 is hydrophobic, the authors thought a water molecule was the most probable cause. This peak was initially assigned to a water molecule, but it was very unstable, especially when using anisotropic atomic displacement parameters. The *R* value was 0.035, but (Δ/σ)_max_ became 6.23 for *U* ^11^ of the oxygens of water. The peak should be considered as a ghost peak. No benzene was found in the sinusoidal channel. The final *R* value was 0.036 using the 3568 observations with |*I*| ≥ 2 σ(*I*) and also 0.057 for all 4998 reflections, and (Δ/σ)_max_ was 0.001. ∑*w*||*F*_o_| − |*F*_c_||^2^ was minimized; *w* = 1/[σ^2^(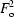) + (0.0793*P*)^2^ + 0.0184*P*], where 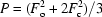, and the final goodness-of-fit parameter (*S*) was 1.04, including anisotropic atomic displacement parameters. The final difference map indicated +0.89 (1) (which is the peak discussed above) and −0.42 e Å^−3^. The positions of the H atoms were calculated and not refined in the calculations. All calculations were performed using the *APEX*II system (Bruker AXS). Table 1 ▶ lists the details of the crystal diffraction analysis.

## 3. Results and discussion

### 3.1. Framework geometry of benzene7.2-silicalite-1

**Figure 5:**
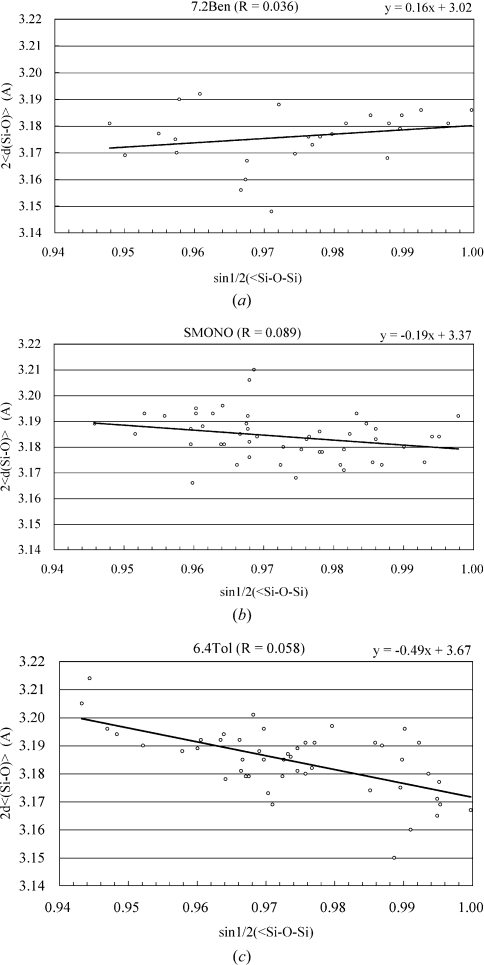
Scatter diagram of 〈*d*(Si—O)〉, plotted as a function of sin 1/2(∠SiOSi) in (*a*) 7.2Ben, (*b*) SMONO and (*c*) 6.4Tol.

Various distances and angles determined in this work for benzene7.2-silicalite-1 (labeled as 7.2Ben) are summarized in Table 2 ▶, along with the corresponding values for toluene6.4-ZSM-5 (labeled as 6.4Tol; Nishi *et al.*, 2005 ▶) and simply prepared monoclinic H-ZSM-5 (labeled as SMONO; Kamiya *et al.*, 2010 ▶). The range of the average Si—O—Si angles in this work for 7.2Ben was similar to those of 6.4Tol and SMONO. The SMONO framework was nearly identical to that of monoclinic H-ZSM-5 (labeled as MONO; van Koningsveld, Jansen & van Bekkum, 1990 ▶), and the 6.4Tol framework structure was similar to that of *p*-xylene8.0-ZSM-5 (labeled as PARA; van Koningsveld, Tuinstra, van Bekkum & Jansen, 1989 ▶). Fig. 5 ▶ shows a scatter diagram of 〈*d*(Si—O)〉 as a function of the Si—O—Si angle, along with the equation of each regression line with an *R* value. The absolute value of the slope of the regression line indicates the stress of each framework structure. The equations of the regression lines of PARA, MONO and high-temperature orthorhombic H-ZSM-5 (labeled as ORTHO; van Koningsveld, 1990 ▶) were also calculated from their work (van Koningsveld, Tuinstra, van Bekkum & Jansen, 1989 ▶) shown in Fig. 3 ▶, and were as follows
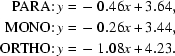

            

The SMONO framework structure stress (slope = −0.19) was similar to that of MONO (slope = −0.26), and the 6.4Tol (slope = −0.49) framework stress was similar to that of PARA (slope = −0.46). However, the 7.2Ben (slope = 0.16) framework structure stress was very different from these, and its absolute value was similar to those of SMONO and MONO. In other words, the framework stress of 7.2Ben was very low.

### 3.2. Packing of benzene in benzene7.2-silicalite-1

#### 3.2.1. Location of benzene in silicalite-1

**Figure 6:**
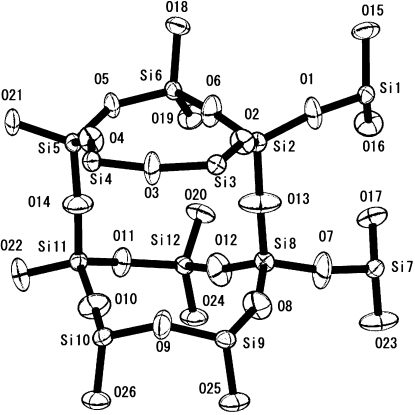
Asymmetric unit of silicalite-1 using the space group *Pnma*.

**Figure 7:**
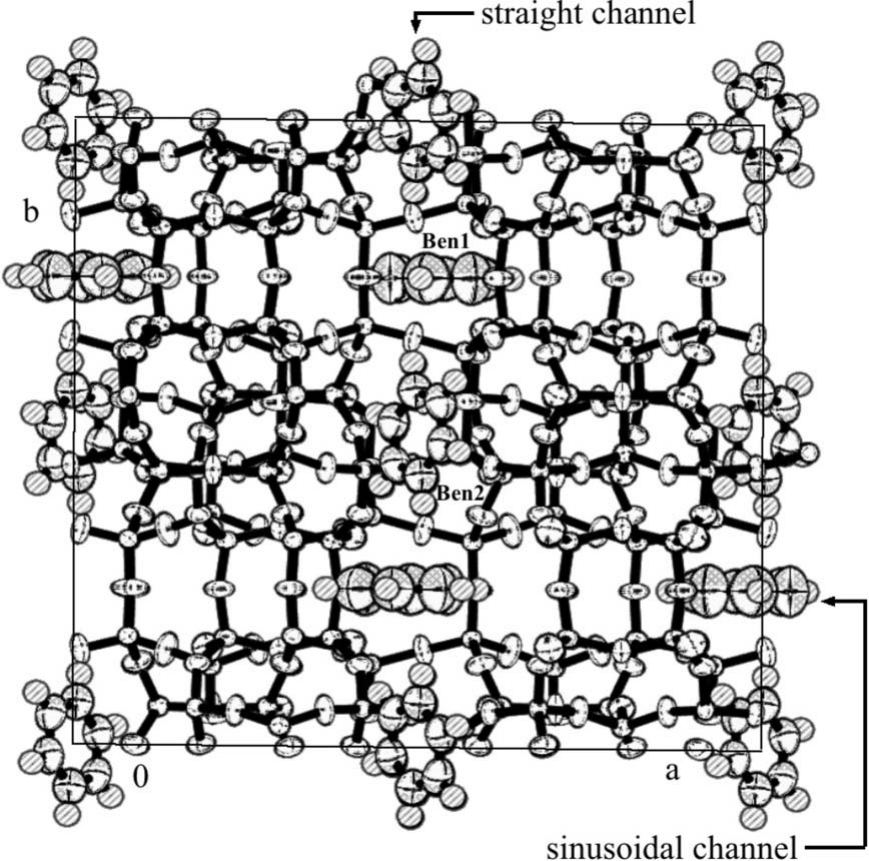
Packing view of benzene7.2-silicalite-1 along the *c* axis.

An asymmetric unit of the silicalite-1 framework is shown in Fig. 6 ▶, and the packing of benzene is shown in Figs. 7 ▶ and 8 ▶. Benzene-to-framework distances of less than 3.7 Å are shown in Table 3 ▶.

Two independent benzene molecules (Ben1 and Ben2) were located in the silicalite-1. Ben1 was at the intersection of the straight channels and the sinusoidal channels and its ring lies on the mirror plane and it is therefore parallel to the *ac* plane. This is the first example of the flat orientation of an aromatic compound parallel to the *ac* plane at any intersection. Ben2 was in the middle of the straight channel. This is the first reported single-crystal X-ray observation of an aromatic hydrocarbon in the straight channel. Ben2 is more tightly packed, as can be seen from Table 3 ▶ and the small *U*_eq_ value in the supplementary material. No benzene molecules were located in the sinusoidal channel. Powder diffraction was also utilized to investigate benzene packing in the ZSM-5 framework (Goyal *et al.*, 2000 ▶; Taylor, 1987 ▶); Goyal, Fitch & Jobie showed that benzene molecules were located at the intersection and in both the straight channel and the sinusoidal channel. On the other hand, Taylor showed that benzene molecules were located at the intersection and in the straight channel. Their results were inconsistent with each other and also differed from our results, especially the conformation of benzene at the intersection. Only the results of Mentzen & Lefebvre (1997 ▶) were similar to ours, and their conformations of Ben1 and Ben2 were almost the same as ours. The occupancy factors of Ben1 and Ben2 are 0.87 (1) and 0.93 (1). Hung & Havenga (2000 ▶) mentioned a similar benzene-silicalite-1 structure in the high loading range of benzene, according to FT–Raman observations. The angle between the positive *an* axis and the normal to the benzene ring plane of Ben2 was approximately 41°. This value is similar to those of 6.4Tol and *p*-dichlorobenzene2.6-ZSM-5 (labeled as 2.6PDCB; van Koningsveld, Jansen & De Man, 1996 ▶).

#### 3.2.2. Benzene in the straight channel

Ben1, Ben2 and the straight channel are shown in Fig. 9 ▶. The atomic distances between C23 and H23 of Ben2 and Ben1 are shown in Table 4 ▶. C23 and H23 are the closest carbon and hydrogen atoms of Ben2 to the Ben1 molecule. The Ben1 and Ben2 contact distances were rather short, judging from the C—H bond lengths (∼ 1.0 Å), and the van der Waals radii (H: 1.2 Å and C: 1.7 Å) shown in Table 4 ▶. The space of the straight channel between two intersections was so small for Ben2 that Ben2 had almost no free-space in the straight channel.

#### 3.2.3. Benzene at the intersection of channels

**Figure 8:**
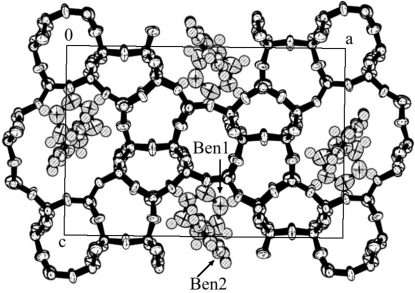
Packing view of benzene7.2-silicalite-1 along the *b* axis.

**Figure 9:**
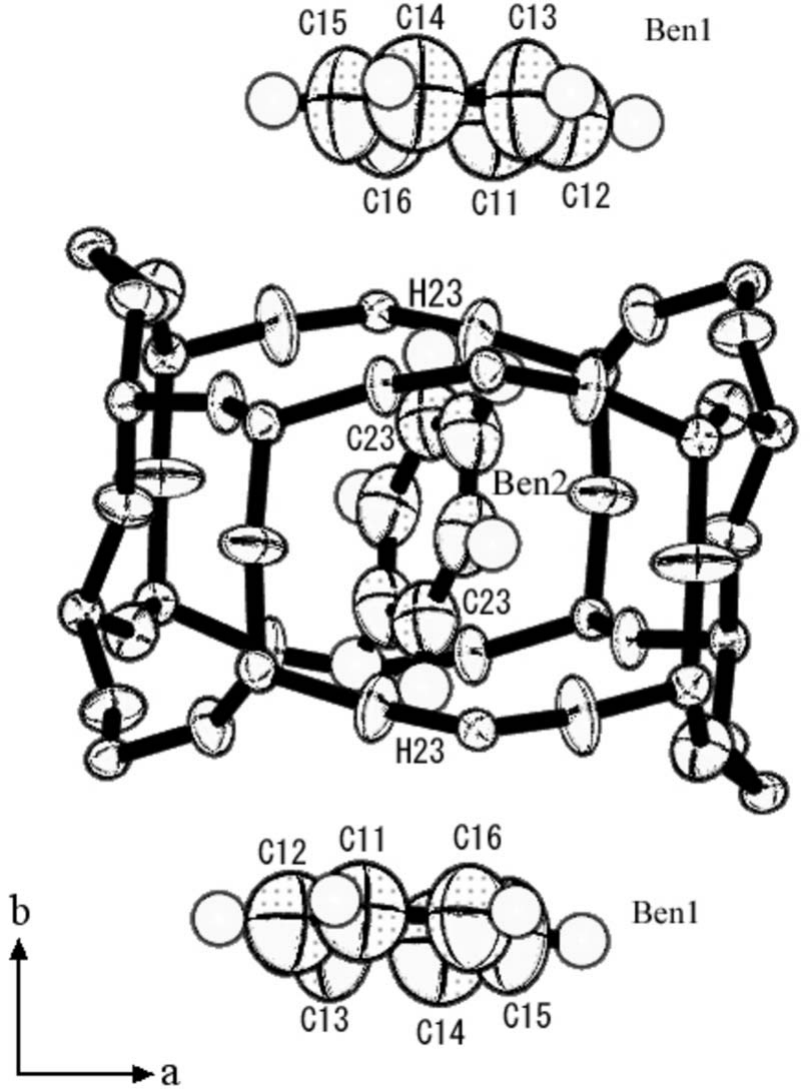
Ben1, Ben2 and the straight channel framework in the benzene7.2-silicalite-1 structure.

**Figure 10:**
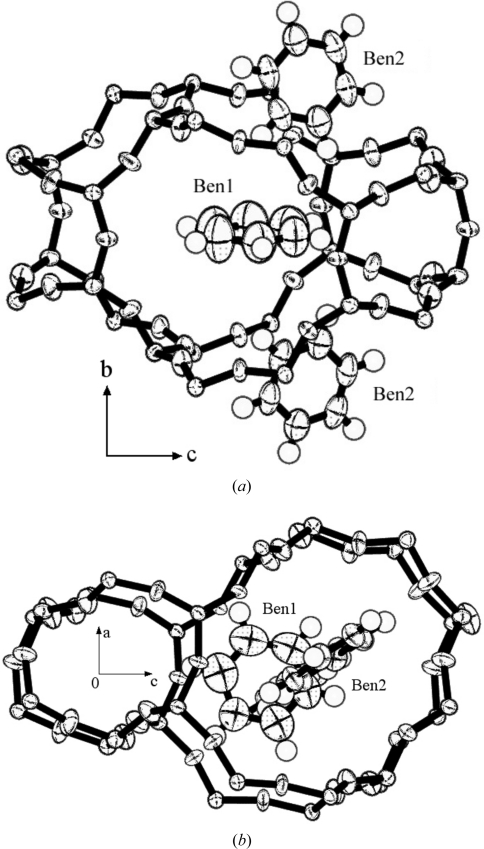
Ben1, Ben2 and the intersection cage in the benzene7.2-silicalite-1 structure: (*a*) along the *a* axis and (*b*) along the *b* axis.

Ben1, Ben2 and the intersection of channels are shown in Fig. 10 ▶. The intersection framework along the *b* axis resembles a 10-oxygen ring pillar, but is actually far more complex. It is constructed from both a 10-oxygen ring and 6-oxygen ring pillar along the *b* axis. The intersection takes the form of a cage, as shown in Fig. 10 ▶. The size of the intersection cage along the *b* axis is the sum of the diameters of six and ten-membered rings (see Fig. 10 ▶ *b*), however, half of the six-membered ring is not part of the intersection cage from Fig. 10 ▶(*a*). The center of the intersection cage is located at (0, *y*, 0.35); see Fig. 8 ▶. The center of Ben1 is approximately the same as the center (0.031, *y*, 0.38) of C11, C16, C14 and C13 from Fig. 9 ▶. Ben1 is located at the mirror plane almost at the center of the intersection cage (Fig. 10 ▶ *a*).

### 3.3. Deformation of the ten-membered ring in benzene7.2-silicalite-1

**Figure 11:**
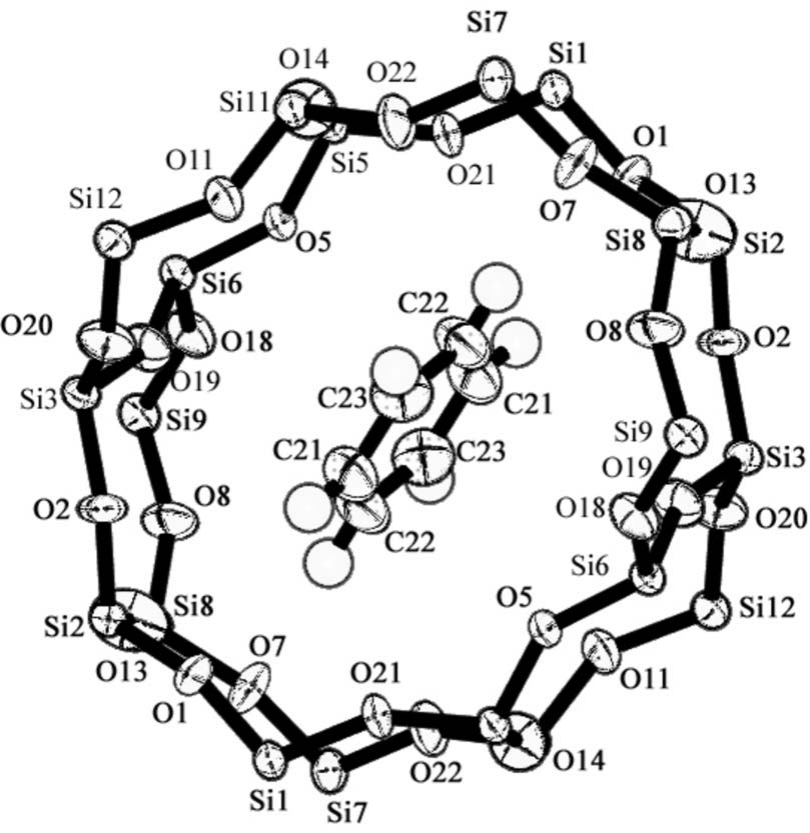
Ben2 and the straight channel framework in the benzene7.2-silicalite-1 structure along the *b* axis.

Ben2 and the straight channel framework in the benzene7.2-silicalite-1 structure is shown in Fig. 11 ▶, and the O—O diagonal distances in the ten-membered rings in the straight channel and sinusoidal channel are shown in Table 5 ▶. The double ten-membered rings in the straight channel became so elliptical that the O1—O7 distance (*l*) was the longest and the O5—O11 distance (*s*) was the shortest. The ratio *l*/*s* was 1.228 because the benzene molecule (Ben2) was located in the straight channel, as shown in Figs. 9 ▶ and 11 ▶. On the other hand, the PDCB (2.6 molecule/u.c.; van Koningsveld, Jansen & De Man, 1996 ▶) was not located in the straight channel, but at the channel intersection in the MFI-type zeolite. In this case PDCB was located at the intersection, the Cl—Cl axis in PDCB was nearly parallel to the *b* axis, and both Cl atoms partially entered the straight channel so that *l*/*s* became 1.180. The geometry of the sinusoidal channel in 7.2Ben was almost the same as that of 2.6PDCB. Both sinusoidal channels were relatively non-deformed, because there was no benzene or PDCB. Mentzen & Lefebvre (1997 ▶) showed that the straight channel and sinusoidal channel deformation (*l*/*s*) are 1.23 (= 9.1/7.4 Å) and 1.06 (= 8.5/8.0 Å) according to their powder data. These values are very similar to our results, as shown in Table 5 ▶.

### 3.4. Adsorption of benzene in orthorhombic silicalite-1

Benzene cannot easily enter the sinusoidal channel of orthorhombic silicalite-1, because the double ten-membered ring is nearly circular (see Table 5 ▶ and van Koningsveld, Tuinstra, van Bekkum & Jansen, 1989 ▶). Benzene may preferentially diffuse through the straight channels and become trapped at the intersection cage. This step is almost the same as that observed with toluene, *p*-xylene and PDCB. At first, toluene molecules occupy the intersection cage, up to four molecules per unit cell. Additional toluene molecules enter the straight channel. Toluene molecules are forced into the sinusoidal channel by intramolecular repulsion. For benzene, the situation is very different. The benzene molecule is smaller than toluene, *p*-xylene or *p*-dichlorobenzene, and the benzene molecule can rotate in the intersection cage to avoid molecular repulsion. Consequently, it becomes oriented parallel to the *ac* plane (see Fig. 10 ▶). This is why no benzene was observed in the sinusoidal channel.

## 4. Conclusions

(i) A new preparation method was developed by the authors. That is, a monoclinic twin crystal of silicalite-1 was pressed along the +*c* and −*c* crystallographic axes, while the temperature was increased from 313 to 473 K over 30 min and then reduced to room temperature over about 6 h in a furnace. These heating and cooling steps were repeated three times resulting in the preparation of single crystals. After using this preparation method, the orthorhombic benzene7.2-silicalite structure was determined by the X-ray single-crystal method.
(ii) Benzene7.2-silicalite structure analysis indicated that there are two independent benzene molecules per unit cell. One (Ben2) is located in the middle of the straight channel. The other (Ben1) is located at the center of the intersection, and the benzene ring is on the mirror plane at the intersection.
(iii) No benzene was found in the sinusoidal channel.

## Supplementary Material

Crystal structure: contains datablock(s) I. DOI: 10.1107/S0108768111038560/dk5001sup1.cif

Structure factors: contains datablock(s) I. DOI: 10.1107/S0108768111038560/dk5001Isup2.hkl

## Figures and Tables

**Table 1:** Crystal data and refinement details

Crystal data	
Chemical formula	C_5.38_H_5.38_O_24_Si_12_
*M*_r_	791.05
Crystal system, space group	Orthorhombic, *Pnma*
Temperature (K)	296
*a*, *b*, *c* (Å)	19.920 (12), 19.880 (13), 13.386 (9)
*V* (Å^3^)	5301 (6)
*Z*	8
*D_x_*	1.982
Radiation type	Mo *K*α
μ (mm^−1^)	0.69
Crystal size (mm)	0.26 × 0.14 × 0.12
	
Data collection	
Diffractometer	Bruker APEX II
Absorption collection	Analytical
*T*_min_, *T*_max_	0.936, 0.946
No. of measured, independent and observed [*I* > 2σ(*I*)] reflections	50 699, 4998, 3568
*R*_int_	0.054
θ_max_ (°)	25.4
	
Refinement	
Refinement on	*F*^2^
*R*[*F*^2^ > 2σ(*F*^2^)], *wR*(*F*^2^), *S*	0.036, 0.130, 1.04
No. of reflections	3568
No. of parameters	380
No. of restraints	1
Δρ_max_, Δρ_min_ (e Å^−3^)	0.89, −0.42

Computer programs used: *XSCANS* (Bruker, 1998 ▶), *SHELXTL*, *SHELXS*97, *SHELXL*97 (Sheldrick, 2008 ▶).

**Table 2:** Comparison of the framework geometry in benzene7.2-silicalite-1 (= 7.2Ben) and simple method of monoclinic ZSM-5 (= SMONO) and toluene6.4-ZSM-5 (= 6.4Tol)

	7.2Ben	SMONO	6.4Tol
O—Si—O range (°)	107.5–111.4 (2)	106.6–111.7 (3)	106.9–112.1 (2)
Average O—Si—O	109.5	109.5	109.5
Si—O range (Å)	1.570–1.601 (2)	1.573–1.615 (5)	1.568–1.614 (4)
Range of average Si—O/SiO_4_	1.578–1.593	1.583–1.599	1.576–1.600
Si—O—Si range (°)	142.8–177.1 (3)	142.1–172.4 (5)	141.2–177.3 (3)
Range of average Si(OSi)_4_	149.0–168.2	148.1–160.2	149.7–162.1

**Table 3:** Benzene to silicalite-1 framework distances (Å) less than 3.70 Å

Ben1 framework		Ben2 framework		Ben2 framework	
C11—O24	3.62	C21—O1	3.45	C22—O1	3.43
C12—O24	3.54	C21—O2	3.42	C22—O2	3.65
C13—O26	3.64	C21—O5	3.44	C22—O5	3.58
C16—O25	3.60	C21—O19	3.65	C22—O13	3.67
		C21—O21	3.33	C22—O19	3.65
				C22—O21	3.29

**Table 4:** Atomic distances (Å) between C23 or H23 of Ben2 and Ben1

		Ben1					
		C11	C12	C13	C14	C15	C16
Ben2	C23	3.76	4.35	4.91	4.95	4.45	3.82
	H23	3.00	3.79	4.49	4.54	3.91	3.07

**Table 5:** Comparison between the results of this work (7.2Ben) and 2.6PDCB for pore opening (diagonal O—O distance, Å: e.s.d. = 0.006 Å) in ten-membered ring in orthorhombic *Pnma*

	7.2Ben (this work)	2.6PDCB
Straight channel		
O1—O7	9.033	8.894
O2—O8	8.485	8.415
O20—O18	7.693	7.971
O11—O5	7.358	7.534
O22—O21	8.241	8.081
*l*/*s*	1.228	1.180
		
Sinusoidal channel		
O1—O2	8.023	8.002
O15—O20	8.246	8.292
O26—O24	8.020	8.049
*l*/*s*	1.028	1.036
O4—O5	8.138	8.062
O17—O18	7.978	7.954
O23—O25	8.383	8.375
*l*/*s*	1.051	1.053

## Footnotes

1 Supplementary data for this paper are available from the IUCr electronic archives (Reference: DK5001). Services for accessing these data are described at the back of the journal.
